# Supplementary material for: IgG-Based Bispecific Anti-CD95 Antibodies for the Treatment of B Cell-Derived Malignancies and Autoimmune Diseases
Source: Cancers (Basel). 2022 Aug 16;14(16):3941. doi: 10.3390/cancers14163941 (PMC9405798; doi:10.3390/cancers14163941)
Supplement: Supplementary file 1 [file cancers-14-03941-s001.zip › cancers-1858139-supplementary.pdf]

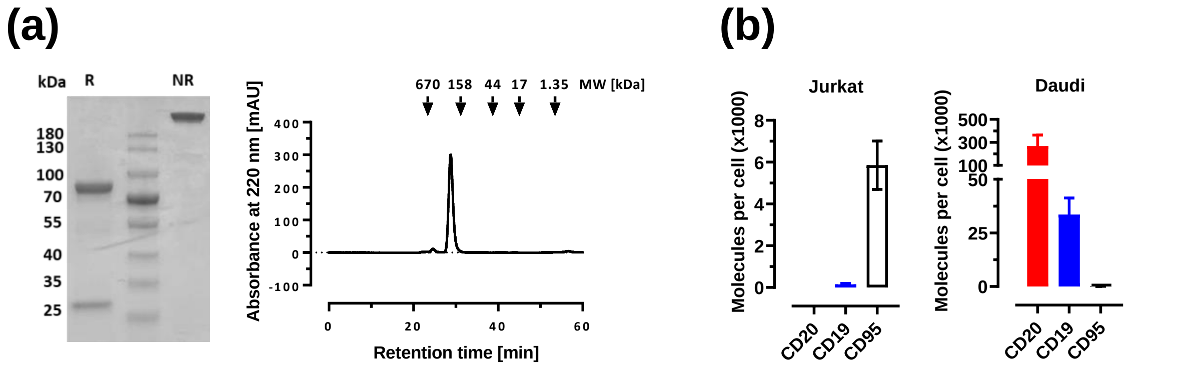

Figure S1. Biochemical characterization of CD95xMOPC and quantitative analysis of antigen expression on Jurkat and Daudi cells. **(a)** SDS-PAGE (left) and analytical size exclusion chromatography (SEC) (right) of the CD95xMOPC molecule. R: reduced; NR: non-reduced. (**b)** The expression of CD95, CD20 and CD19 on Jurkat (left) and Daudi (right) cells was calculated using the flow cytometry-based Qifikit system. Mean±SD, n=3.

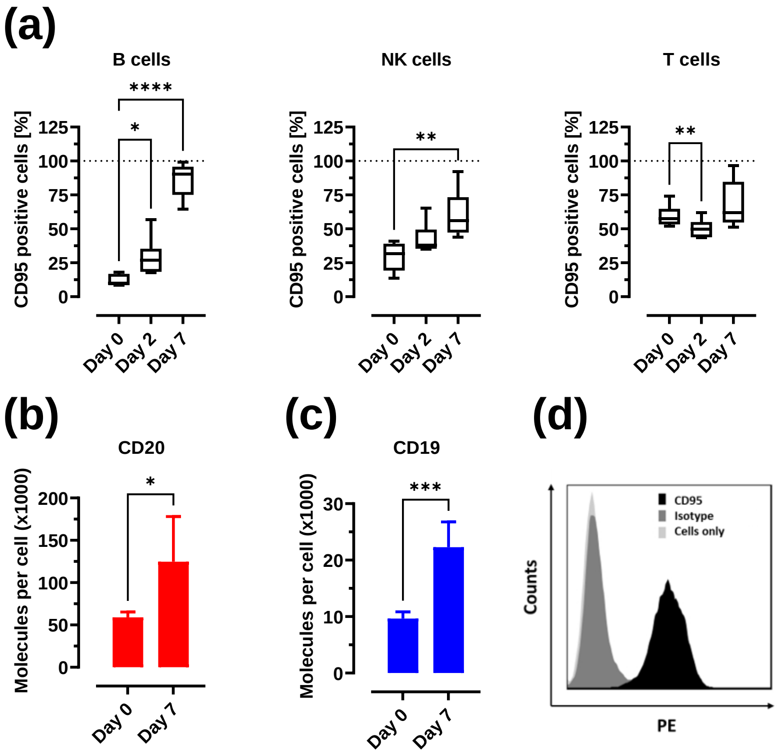

Molecules per cell (x1000)	Day 0	Day 7
**CD20**	59.0±6.21	124±53.3
**CD19**	9.65±1.21	22.3±4.48

Figure S2. Antigen expression on resting and activated lymphocytes. **(a-c)** To activate B cells, PBMCs were stimulated for 7 days with 0.1 µM of ODN2006. **(a)** The expression of CD95 on different lymphocyte populations was verified at different time points via flow cytometry. Boxplot and whiskers from six different donors. Statistics were calculated with One-Way ANOVA. Day 0 versus day 2 or day 7. **(b)** and **(c)** The antigen density of CD20 (A) and CD19 (B) on the cell surface of resting or activated B cells was calculated using the flow cytometry-based Qifikit system. The corresponding analysis is presented in the table under the graph. Mean±SD from six different donors. Statistics were calculated using a paired t-test. **(d)** Expression of CD95 on LX-1 hepatic stellate cells.
